# Deletion patterns, genetic variability and protein structure of *pfhrp2* and *pfhrp3*: implications for malaria rapid diagnostic test in Amhara region, Ethiopia

**DOI:** 10.1186/s12936-022-04306-3

**Published:** 2022-10-08

**Authors:** Irene Molina - de la Fuente, Mulat Yimar, Luz García, Vicenta González, Arancha Amor, Melaku Anegagrie, Agustín Benito, Javier Martínez, Marta Moreno, Pedro Berzosa

**Affiliations:** 1 Department of Biomedicine and Biotechnology, School of Pharmacy, University of Alcalá, Alcalá de Henares, Madrid, Spain; 2 Malaria and Neglected Diseases Laboratory, National Centre of Tropical Medicine, Institute of Health Carlos III, Madrid, Spain; 3 Public Health and Epidemiology Research Group, School of Medicine, University of Alcalá, Alcalá de Henares, Madrid, Spain; 4 College of Medicine and Health Sciences, Bahir Dar University, Bahir Dar, Ethiopia; 5 CIBERINFECT - CIBER Infectious Diseases (ISCIII), Madrid, Spain; 6 Mundo Sano Foundations, Institute of Health Carlos III, Madrid, Spain; 7 Department of Infection Biology, London School of Hygiene and Tropical Medicine, London, UK

**Keywords:** Malaria, Ethiopia, *Plasmodium falciparum*, *pfhrp2*, Rapid diagnostic test, Deletions

## Abstract

**Background:**

Although rapid diagnostic tests (RDTs) play a key role in malaria-control strategies, their efficacy has been threatened by deletion and genetic variability of the genes *pfhrp2/3*. This study aims to characterize the deletion, genetic patterns and diversity of these genes and their implication for malaria RDT effectiveness, as well as their genetic evolution in the Amhara region of Ethiopia.

**Methods:**

The study included 354 isolates from symptomatic patients from the Amhara region of Ethiopia who tested positive by microscopy. Exon 1–2 and exon 2 of genes *pfhrp2* and -*3* were amplified, and exon 2 was sequenced to analyse the genetic diversity, phylogenetic relationship and epitope availability.

**Results:**

The deletion frequency in exon 1–2 and exon 2 was 22 and 4.6% for *pfhrp2*, and 68 and 18% for *pfhrp3*, respectively. Double deletion frequency for *pfhrp2* and *pfhrp3* was 1.4%. High genetic diversity, lack of clustering by phylogenetic analysis and evidence of positive selection suggested a diversifying selection for both genes. The amino-acid sequences, classified into different haplotypes, varied widely in terms of frequency of repeats, with novel amino-acid changes. Aminoacidic repetition type 2 and type 7 were the most frequent in all the sequences. The most frequent epitopes among protein sequences were those recognized by MAbs 3A4 and C1-13.

**Conclusion:**

Deletions and high amino acidic variation in *pfhrp2* and *pfhrp3* suggest their possible impact on RDT use in the Amhara region, and the high genetic diversity of these genes could be associated with a diversifying selection in Ethiopia. Surveillance of these genes is, therefore, essential to ensure the effectiveness of public health interventions in this region.

**Graphical Abstract:**

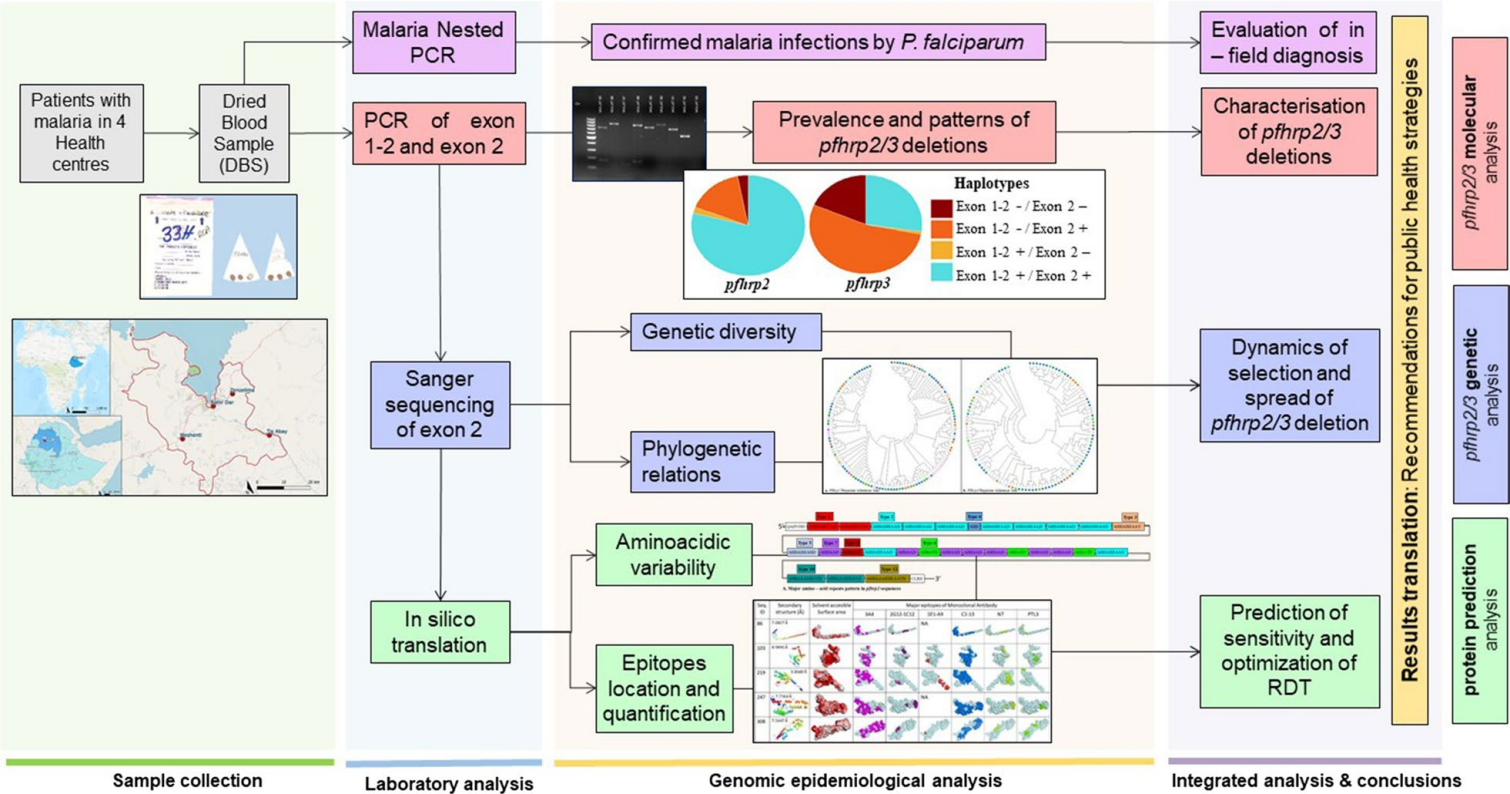

**Supplementary Information:**

The online version contains supplementary material available at 10.1186/s12936-022-04306-3.

## Background

Malaria, the cause of 627,000 of deaths in 2020, is concentrated in Africa, with the majority of cases being caused by *Plasmodium falciparum* [1]. According to World Health Organization (WHO), malaria-control programmes are expected to rely on test-and-treat strategies, where the effectiveness of diagnosis is essential to ensure a prompt treatment [2]. In this regard, rapid diagnostic tests (RDTs) are becoming increasingly popular as they provide a simple and cheap diagnosis [3].

RDTs are based on detection of specific parasitic proteins [4], and the majority of RDTs for *P. falciparum* rely on the detection of *P. falciparum* histidine rich protein-2 (PfHRP2), which reports cross-reactions with *P. falciparum* histidine rich protein-3 (PfHRP3) [5, 6]. PfHRP2 is the preferred protein for RDTs due to its stability, specificity and abundant expression during the blood stage [7].

The *pfhrp2* and *pfhrp3* genes comprise two exons, namely exon 1–2, which contains signal and cleavage sequences, and exon 2, which is the encoding sequence [8]. Deletions in *pfhrp2* and *pfhrp3* could be the cause of misdiagnosis and of false-negative *P. falciparum* results in HRP2-based RDTs [9, 10]. This could have an impact on delay or lack of treatment, with consequences for both the patient and for the maintenance of malaria transmission [11, 12]. In addition, *pfhrp2* and *pfhrp3* are highly variable genes with tandem repetitions [13, 14].

Variations in amino acid repeat motifs have also been reported to be a factor that may modify the effectiveness of HRP2-based RDTs [15, 16]. In this regard, although the main epitopes of PfHRP2 have been identified previously [17], the protein structures of PfHRP2 and PfHRP3 are still unknown, which complicates prediction of the epitope availability or the effect of polymorphisms [18, 19].

The dynamics of the evolution of these genes is not well understood. Thus, although there is evidence of selective pressure on *pfhrp2* and *pfhrp3* [20, 21], including in Ethiopia [22], multiple origins of parasites with deletions have also been reported suggesting spontaneous and multiple deletion events [23, 24].

Ethiopia is the most populous country in eastern Africa and reported approximately 260,000 malaria cases in 2019, with 5626 deaths [25]. However, it is considered to be a country in the process of consolidating malaria control, with this disease being heterogeneously distributed [26]. Amhara has a low-moderate malaria transmission, with a *Plasmodium sp.* prevalence of 0.8% in 2015 [27].

HRP2-based RDTs were implemented in Ethiopia in 2004 and have been used since 2010 as evidence for treatment as part of a test-and-treat policy [28]. Since then, however, deletions in *pfhrp2* and *pfhrp3* genes have been reported in some regions of Ethiopia [22, 29, 30], and neighbouring countries, such as Kenya [31] and Eritrea [10], thereby jeopardizing the efficacy of test-and-treat policies and efforts made by National Malaria Programmes in their implementation. As such, an increased understanding of the status of these genes and their genomic dynamics could help us to better understand their dynamics and design future guidelines. Moreover, there are no studies in Ethiopia focussing on the structural diversity of HRP2 and HRP3 proteins.

In light of the above, the aims of this study were to characterize the deletion prevalence and patterns of exons 1–2 and 2 of the *pfhrp2* and *pfhrp3* genes in the Amhara region and to study their genetic diversity, using the parameters diversity and haplotype networks, along with the amino-acid sequence variations in exon 2. The predicted sensitivity of HRP2-based RDT was also assessed using a theoretical model and the frequency and localization of epitopes on predicted protein structure models. Also assessed was the possible force driving selection and the evolution of these genes by performing a phylogenetic and genetic evolution analysis.

## Methods

### Sample collection and study area

**Fig. 1:**
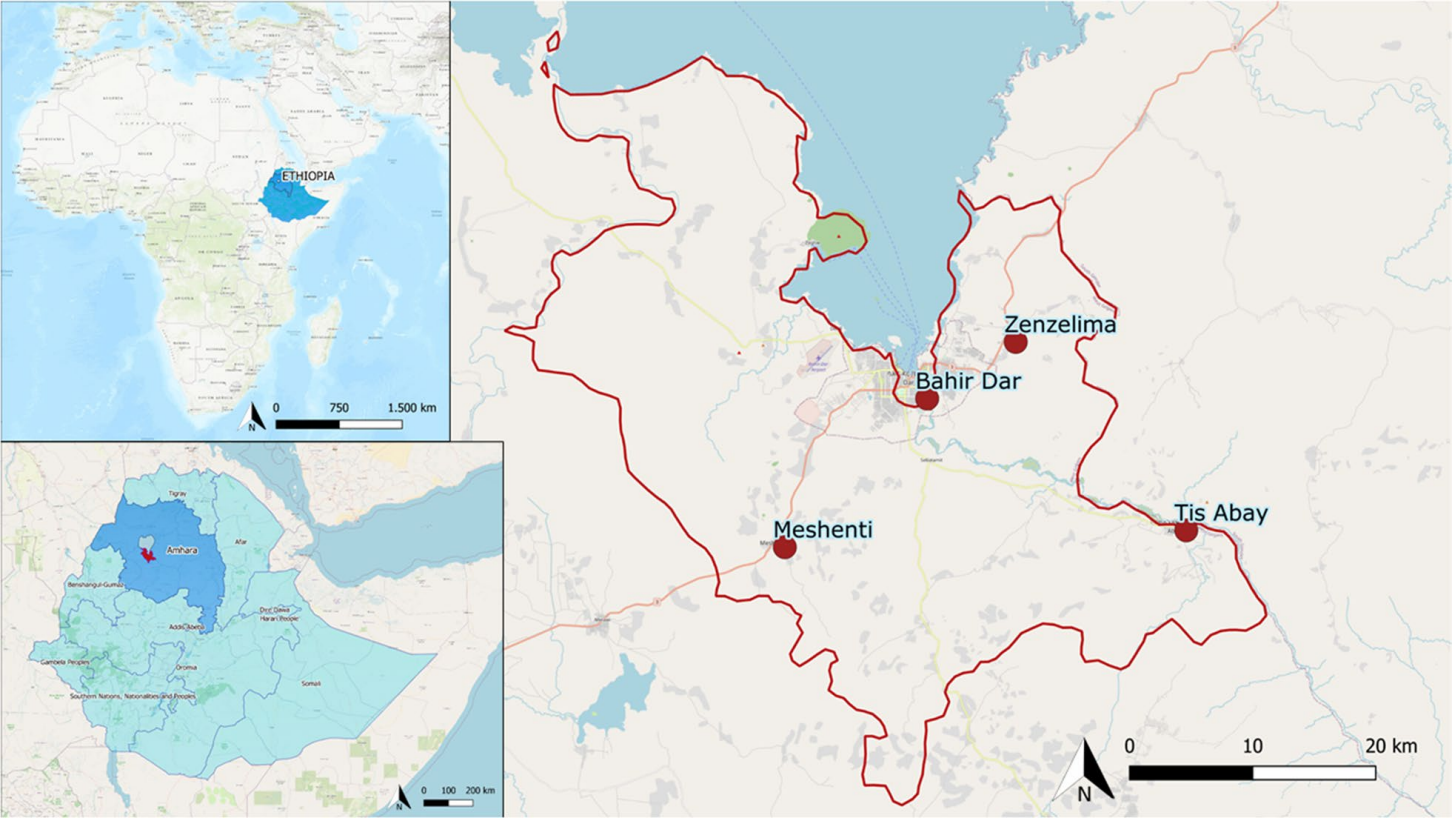
Map of collection sites in Ethiopia. The red dots correspond to the location of the four cities’ health centres where samples were collected

The study was conducted in the Amhara region, Bahir Dar Department, Ethiopia (East Africa). During the malaria transmission season in 2014 (September to December), samples from symptomatic malaria infections from outpatients at health facilities were collected. Febrile or otherwise malaria-symptomatic patients aged ≥ 16 years with positive *P. falciparum* diagnosis by microscopy were eligible to participate. Samples were collected at four health facilities: Bahir Dar city, the principal city of the region near to Tana Lake (population: 170,000); Tis Abay, a town surrounded by farming areas close to the Blue Nile Falls waterfall; Meshenti, a medium-sized city to the southwest of Bahir Dar city; and, Zenzelima, a rural and surrounding area to the northeast of Bahir Dar city (Fig. 1). This region has a sub-tropical highland oceanic climate, with temperatures ranging between 18 and 24 °C (an average temperature of 20 °C), and abundant precipitation concentrated in the rainy season (rainfall around 1850 mm per year).

Blood samples were taken on Whatman 903TM paper (GE Healthcare Bio-Sciences Corp.) by finger prick and stored in double zip-lock plastic bags with silica gel at − 20 °C, and while they were being analysed they were stored at 4 °C. They were then transported to the National Centre for Tropical Medicine, Institute of Health Carlos III (Madrid, Spain) for molecular and genetic analysis.

### DNA extraction and molecular diagnosis of *Plasmodium* sp.

DNA was extracted from dot blood spots on filter paper using the chelex–saponin method [32]. A 5-mm diameter punch containing 10 μL of blood was used [33]. Malaria molecular diagnosis was performed on all samples using nested multiplex PCR targeting the 18S small sub-unit ribosomal RNA (ssRNA) to confirm *P. falciparum* infections [34, 35]. Amplification of other *P. falciparum* genes (*pfdhfr*, *pdhps*, *pfmdr1*, *pfcrt*) was performed to ensure that the samples were free of PCR-inhibiting elements and thus confirm that when the *pfhrp2* and *pfhrp3* genes did not amplify it was due to a true deletion and not inhibition.

### *pfhrp2* and *pfhrp3* gene deletions

**Fig. 2:**
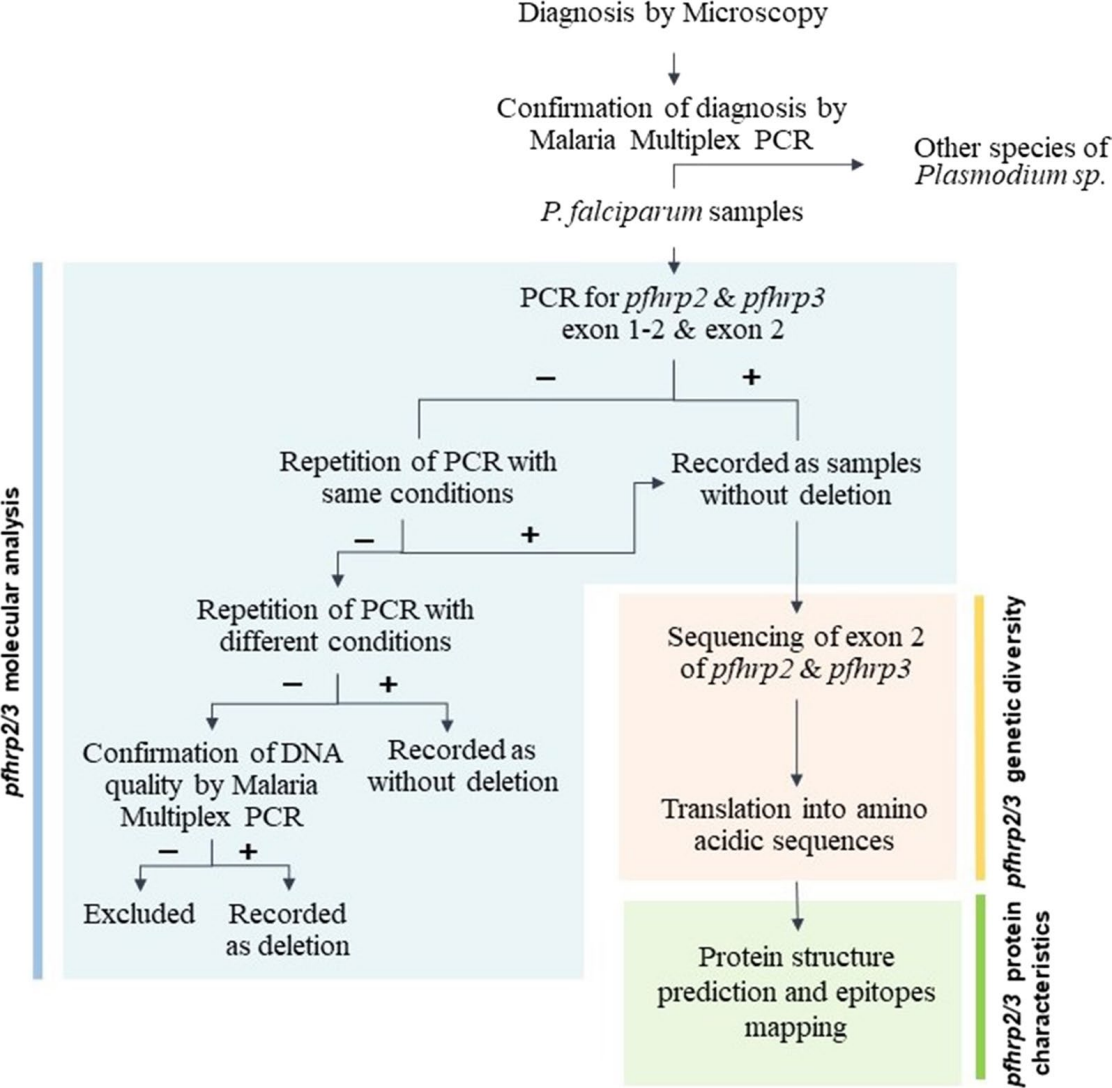
Methodological flow scheme. Microscopy diagnosis was confirmed by nested multiplex PCR, distinguishing *P. falciparum*, *Plasmodium vivax*, *Plasmodium Ovale*, and *Plasmodium malaria*. Four independent PCRs were run for the *P. falciparum* samples to detect deletions in exon 1–2 and exon 2 of *pfhrp2* and *pfhrp3*. The deletion of any exon was confirmed by an absence of amplification after three PCR repetitions and the confirmation of maintained DNA quality. Samples that could not be confirmed for DNA quality were excluded from the analysis for that gene. Subsequent data analysis for the combined results was performed only with those samples included in the independent analysis for both genes. Additionally, a sub-sample lacking the deletion for exon 2 of both genes was sequenced and the genetic diversity and variation in amino-acid sequences analysed. Finally, a sub-sample of these sequences was used to predict the protein structure model and to locate the epitopes in the structure

Amplification of exon 2 of *pfhrp2* and *pfhrp3* was performed by semi-nested PCR following a previously described procedure [10] and amplification of exon 1–2 of *pfhrp2* and *pfhrp3* was performed following a protocol with slight modifications [36], such as a lower annealing temperature (55 °C) and more cycles for both PCRs (35 cycles). A summary of the workflow is described in Fig. 2. PCR amplicons were separated and visualized on a 2% agarose gel stained with Pronasafe (Pronadisa SA, Spain). If no amplification was observed, the PCR experiment was repeated, and, only for exon 2 of both genes, if the result was still negative, it was repeated a third time with a lower elongation temperature (60 °C) [37]. If there was no amplification in any of the experiments, the maintenance of DNA quality was confirmed by nested multiplex PCR, and if amplification was observed, the result was considered to be a deletion event.

Samples with deletion in exon 1–2 but not in exon 2, or vice versa, were also amplified using the forward primer for exon 1–2 and the reverse primer for exon 2, and the previously reported PCR protocols for exon 2 of both genes. Samples that did not amplify with that PCR were assumed to have had some part of the exon that did not amplify also not previously deleted. *Plasmodium falciparum* clone 3D7 was used as a positive control in all PCRs, with clone Dd2 as negative control for the *pfhrp2* gene amplification. This clone contains a pfhrp2 gene deletion. Similarly, clone HB3, with *pfhrp3* deleted, was used as negative control for the *pfhrp3* gene amplification.

### Genetic diversity of exon 2 from *pfhrp2* and *pfhrp3* genes

#### Sequencing and genetic analysis of exon 2 from *pfhrp2* and *pfhrp3*

A random sub-sample of PCR-positive samples for both genes (*pfhrp2* and *pfhrp3*) was sequenced. The PCR products were purified using Ilustra Exoprostar 1-step (GE Healthcare Life Sciences) following the manufacturer’s instructions. Samples were sequenced in both directions using forward and reverse primers for the second PCR of exon 2 at a concentration of 6 pmol/μL, sequencing using a standard dye terminator in an ABI PRISM 3730 XL Analyser (Big Dye Terminator v3.1 Cycle Sequencing kit).

Bio Edit Sequence Alignment Editor Software v7.1.3.0 was used for sequence editing and solving undetermined sites. Mega7 v7.0.26 was used for multi-sequence alignment with ClustalW tool, a progressive alignment method recommended for data sets with varying degrees of divergence [38]. Polymorphism was then analysed by determining the diversity of haplotypes, nucleotide diversity (π) and genetic diversity (Θ) using DNAsp v6.12.03. Diversity was analysed through pairwise comparison, where gaps/missing presented in each comparison were excluded but only for that comparison. Haplotype networks were constructed using the Median Joining Network method, which is phylogeographical analysis with lack of rooting, in PopART v.1.7. The randomness of the distribution of haplotypes between populations was assessed using the Exact test for population differentiation based on Haplotype Frequencies calculated in Arlequin v3.5.2.2. Neutrality tests, namely Tajima’s D, which is based on allele frequencies, and Fu and Li’s D*, which analyses synonymous and non-synonymous sites, were calculated using DNAsp v6.12.03 as a means of detecting selection.

#### Phylogenetic analysis

The evolutionary relationships of exon 2 *pfhrp2* and *pfhrp3* were assessed for the same sub-sample of nucleotide sequences sequenced for genetic analysis using the Datamonkey server [39]. First, genetic recombination was evaluated using the Genetic Algorithm for Recombination Detection (GARD) tool [40]. GARD is a pre-processing step for selection inference because it groups unique phylogenetic histories for each recombination block. The Branch-site Unrestricted Statistical Test for Episodic Diversification (BUSTED) tool was then applied to detect gene-wide positive selection at at least one site on at least one branch [41].

To assess phylogenetic relationships combined with clustering by geographical location. Phylogenetic trees were performed using Bayesian Inference by MrBayes v3.2.7a and Maximum Likelihood by Mega7 v7.0.26 as inference methods to estimate evolutionary distances between all sequences. Both methods used substitution models GTR + T + I, where missing data or alignments gaps were not considered, so loci with gaps or missing data were eliminated of the analysis. In this analysis, 33 homologous sequences of each gene from countries bordering Ethiopia and other regions of Sub-Saharan Africa were included, published in GenBank (Additional file 1).

### Study of amino acid sequences and protein structure

#### Variation in amino acid repeats patterns

All the sequenced samples were used to amino acid repeats characterization with a number according to Baker et al. [15] and recent repeats characterized in Kenya [42]. When two or more amino acid repeats overlapped, only the largest repeat was counted. Analysis of the frequency and organization of amino acid repeats was carried out with a custom code using Python 3.9 Software (Additional file 2).

#### In silico translation of exon 2 of *pfhrp2* and *pfhrp3*

Prediction of the secondary and tertiary structure of the protein was performed using two different platforms to obtain more consistency in the results: (1) RaptorX (http://raptorx.uchicago.edu); and (2) Protein Homology/analogy Recognition Engine v2.0 (PHYRE2) (http://www.sbg.bio.ic.ac.uk/phyre2/html/page.cgi?id=index). Both analyses applied a hierarchical approach with a combination of derived-template and de novo protein prediction to generate full-chain models. Firstly, amino acidic sequences were submitted in PHYRE2 for a batch-processing, then sequences with higher quality and confidence were selected for following analysis, structure prediction by RaptorX. RaptorX showed the best results for secondary and tertiary structures. Model quality was estimated using the RMSD (root mean square deviation) value and the model with lowest RMSD was selected as this indicates the deviation observed between the native and predicted positions [43]. Additionally, PHYRE2 was used for structure-based function prediction, according to the alignments.

#### Description and localization of epitopes in the protein

The presence of 11 linear epitopes, previously described as the epitopes for optimal monoclonal antibodies potentially used in HRP2-based RDTs, as information about mAbs used in commercial RDT kits is unavailable, was confirmed in protein sequences and the frequency of each epitope per sequence was analysed. The median repetition of each epitope, and its frequency among the sequences, was calculated. The PyMOL2 molecular graphic system v.2.5 (https://pymol.org/2/) was used to locate the epitopes in the predicted tertiary structure and APBS [44] and PDB2PQR [45] were used to calculate the Solvent Accessible Surface area.

## Results

### Deletions in *pfhrp2* and *pfhrp3* genes

A total of 354 filter paper samples were collected from symptomatic patients with malaria (diagnosed by microscopy) from Tis Abay (N = 200), Bahir Dar (N = 81), Zenzelima (N = 35), and Meshenti (N = 38). Of these, 52 samples were excluded due to low DNA quality. *Plasmodium falciparum* was confirmed in most of the samples (294/302; 97.35%), *P. vivax* was identified in 2.65% (8/302) and no mixed infections were detected.

**Fig. 3:**
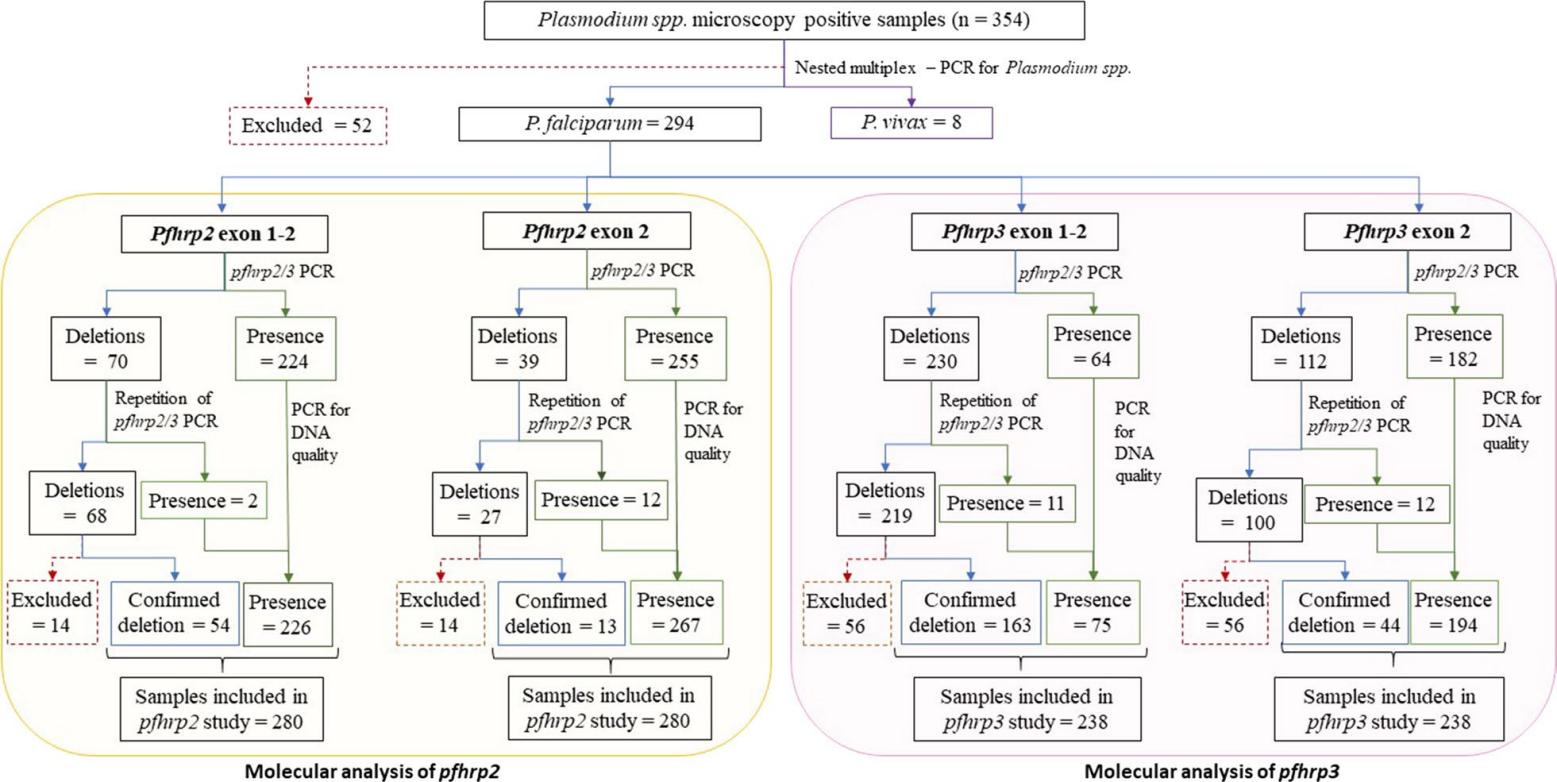
Summary of results of molecular analysis of *pfhrp2* and *pfhrp3.* Figure represents the different results in the independent experiments for exon 1–2 and 2 of both genes. Deletions samples: samples that did not amplify in the first PCR for the exon. Confirmed deletions: samples that did not amplify in three independent PCR experiments for the same specific gene, process called ‘repetition *pfhrp2/3* PCR’ in the figure. Presence: samples that amplified at least one for a specific exon

*Plasmodium falciparum* samples were used for amplification of exon 1–2 and exon 2 of *pfhrp2* and *pfhrp3* genes. After the confirmation of deleted samples (i.e., deletion in *pfhrp2* and/or *pfhrp3* genes) 280 samples were included for the *pfhrp2* analyses and 238 samples for the *pfhrp3* analyses (Fig. 3). Negative samples that could not be confirmed to maintain sufficient DNA quality were excluded from the analysis (n = 5 for *pfhrp2* analysis only, n = 47 for *pfhrp3* analysis only, and n = 9 for both *pfhrp2* and *pfhrp3* analysis). For joint outcomes of both genes, 219 samples were included as they were only samples that were not excluded from any of the analyses.

#### Frequency of deletion in exons 1–2 and 2 of *pfhrp2* and *pfhrp*3 genes

**Fig. 4:**
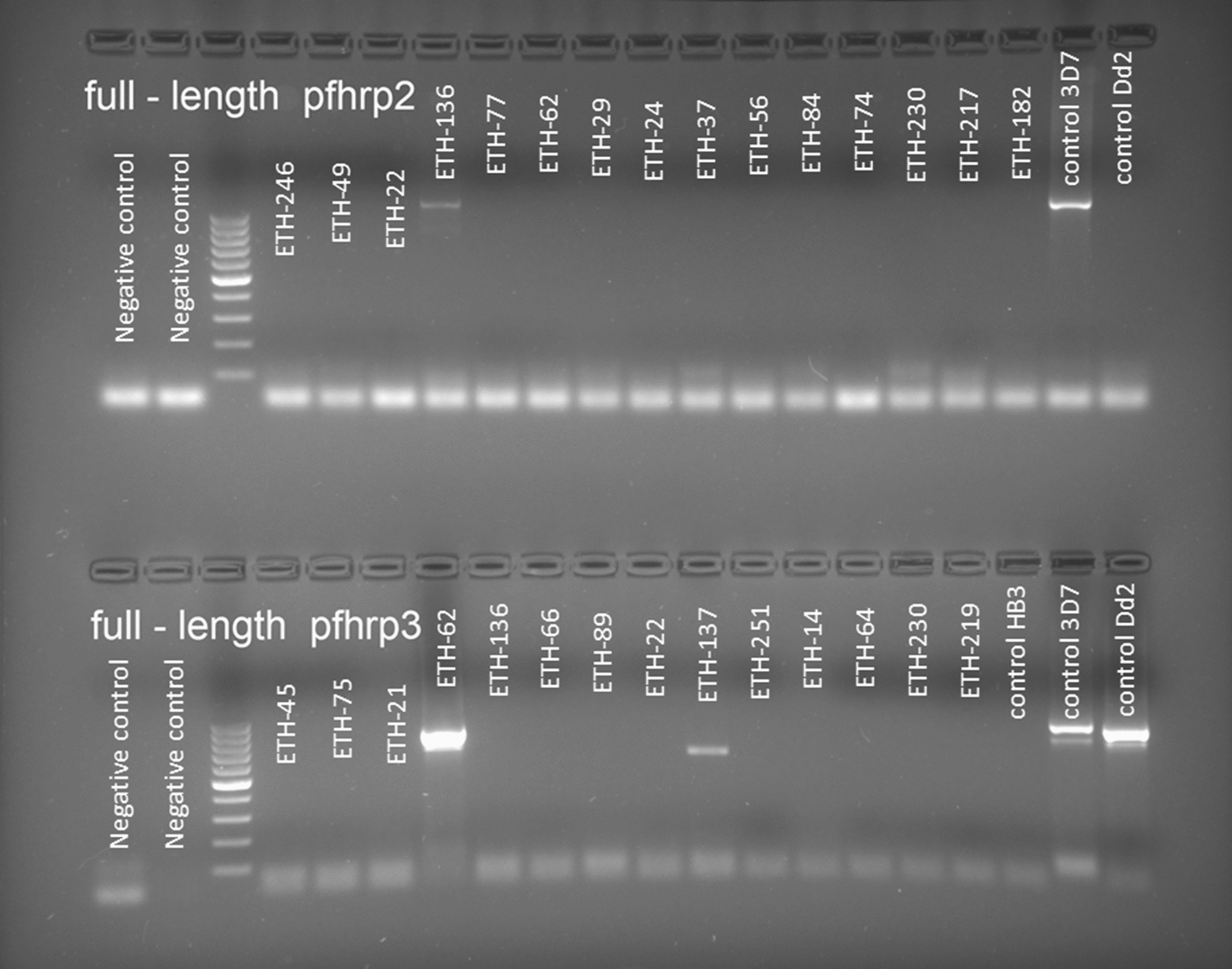
Gel images of the full-length PCR of *pfhrp2* and *pfhrp3*. Electrophoresis gel showed the result of the amplification for PCR combining exon 1–2 and exon 2, the expected fragment was around 900 base pairs as it could be observed in the positives controls placed at the end of the gel: 3D7, Dd2 and HB3. Negative controls for the first and second PCR were placed in the first two wells of the gel

Deletions were detected in exons 1–2 and 2 for both genes, with a higher frequency of deletions in exon 1–2 (19.29% (54/280) for *pfhrp2* and 68.49% (163/238) for *pfhrp3*) than in exon 2 (4.64% (13/280) for *pfhrp2* and 18.49% (44/238) for *pfhrp3*). Candidate partial deletion was considered when only exon 1–2 or 2 have amplified but ‘partial deletions’ could not be confirmed. Full-length PCR was performed for partially deleted candidate samples, but only 5 of 51 of *pfhrp2* and 14 of 123 *pfhrp3* amplified with that PCR showing different fragment sizes than expected (Fig. 4).

*Pfhrp2* presented at least one deleted exon in 21.08% of isolates and 2.86% of them had the *pfhrp2* gene completely deleted (any exon amplified) (Table 1). A high proportion of isolates presented some deletion in *pfhrp3* (73.95%), with complete deletion of this gene being detected in 18.49% of isolates. There were significant differences in the frequency of exon 2 of *pfhrp3* deletion by location (p-value < 0.05), but not for exon 2 of *pfhrp2*. Frequencies of exon 2 of *pfhrp2* deletion are similar between locations but *pfhrp3* deletion showed significant differences by location (Additional file 3).

**Table 1:** Deletion patterns for exon 1–2 and exon 2 of *pfhrp2* and *pfhrp3* genes

	Exon 1–2	Exon 2	F (90% IC)	n (N)
*pfhrp 2*				
Complete deletion	−	−	2.86 (1.49–5.22)	8 (280)
Candidate partial deletion	−	+	16.43 (12.94–20.58)	46 (280)
Candidate partial deletion	+	−	1.79 (0.76–3.86)	5 (280)
Complete gene	+	+	78.93 (74.46–82.82)	221 (280)
*pfhrp 3*				
Complete deletion	−	−	17.64 (13.76–22.29)	42 (238)
Candidate partial deletion	−	+	50.84 (45.32–56.34)	121 (238)
Candidate partial deletion	+	−	0.84 (0.18–2.81)	2 (238)
Complete gene	+	+	30.67 (25.16–36.80)	73 (238)

Exon 1–2 −: samples that did not amplify for exon 1–2; Exon 1–2 +: samples that amplified for exon 1–2; Exon 2 −: samples that did not amplify for exon 2; Exon 2 +: samples that amplified for exon 2

#### Deletion patterns in *pfhrp2* and *pfhrp3*

Overall, double-negative samples (*pfhrp2* and *pfhrp3* completely deleted) were found in 1.37% (3/219) of cases and both complete genes were identified in 30.59% (67/219) (Table 2).

**Table 2:** Frequencies of deletion patterns based on deletion/presence of exon 1–2 and exon 2 of *pfhrp2* and *pfhrp3* genes

*pfhrp 2*		*pfhrp 3*		F (90% IC)	Number of isolates (N = 219)
Exon 1–2	Exon 2	Exon 1–2	Exon 2		
+	+	+	+	30.59 (25.52–36.16)	67
−	−	+	+	0	0
+	+	−	−	13.70 (10.12–18.22)	30
−	+	−	+	8.68 (5.84–12.59)	19
+	−	+	−	0.46 (0.01–2.38)	1
−	−	−	−	1.37 (0.42–3.69)	3

### Genetic diversity of exon 2 of *pfhrp2* and *pfhrp3* genes

A total of 95 *pfhrp2* and 79 *pfhrp3* exon 2 *P. falciparum* sequences were analysed and translated successfully into amino acids (aa). These sequences were submitted to the GenBank database and the homology with the *pfhrp2* and *pfhrp3* genes of *P. falciparum* checked for further analysis. The size of the exon 2 sequence ranged from 400 to 750 bp in *pfhrp2* and 350 to 700 bp in *pfhrp3*, with an average of 210 and 188 amino acids, respectively.

A total of 92 haplotypes were identified in the *pfhrp2* sequences, with 93.68% of sequences being unique (89/95). All the haplotypes shared by more than one sample include only two sequences (Additional file 4). A total of 77 haplotypes were detected for the *pfhrp3* sequences, with two haplotypes shared by two samples from different health centre. All haplotype sequences were submitted to the GenBank database (accession numbers: OL961136–OL961227 and OL961228–OL961304).

The haplotype analysis of *pfhrp2* showed a higher diversity, in terms of haplotype distribution and frequency, than the haplotype network for *pfhrp3*, for which lower distribution for the different haplotypes were observed (Additional file 4). These results are consistent with the genetic diversity analysis, which revealed a higher nucleotide diversity for *pfhrp2* (π = 0.158) than for *pfhrp3* (π = 0.027) (Table 3).

**Table 3:** Genetic diversity of *pfhrp2* and *pfhrp3* by geographical location

Health centre	No. of isolates	S	H	K	π	Θ per site	Θ per sequence
*Pfhrp2* gene							
Overall	95	532	92	63.36	0.158	0.162	135.53
Tis Abay	57	419	55	61.32	0.150	0.147	108.07
Bahir Dar	16	305	16	65.13	0.175	0.180	108.73
Zenzelima	3	532	3	63.36	0.159	0.162	135.54
Meshenti	19	320	19	65.46	0.163	0.151	114.65
*Pfhrp3* gene							
Overall	79	188	77	10.223	0.027	0.083	49.02
Tis Abay	50	140	48	9.01	0.025	0.071	41.75
Bahir Dar	13	91	13	13.41	0.035	0.068	34.29
Meshenti	16	57	16	10.11	0.023	0.049	25.06

Results were obtained using pairwise comparison, 4,465 pairwise comparisons have been performed and the average number of sites analysed by comparison was 405,72, total number of sites were 916

S: number of segregating sites; H: number of different haplotypes; K: average number of nucleotide differences; π: nucleotide diversity; Θ per site: genetic diversity per site; Θ per sequence: genetic diversity per sequence

Test statistics for neutrality were applied to assess the hypothetical evolution of the genes. The neutrality tests for *pfhrp2* were both negative (Fu and Li’s D* = –2.43 and Tajima’s Neutrality Test D = − 0.96), but only Fu and Li’s D* was statistically significant (p-value < 0.05). In contrast, the neutrality tests for *pfhrp3* were both negative (Tajima’s D = − 2.23 and Fu and Li’s D* test statistic = − 6.05) and statistically significant (p-value < 0.05).

### Phylogenetic and evolution analysis

The ClustalW alignment resulted in an alignment of 916 base pairs for *pfhrp2* and 618 sites for *pfhrp3*.

Potential recombination breakpoints between the *pfhrp2* and *pfhrp3* sequences were detected by GARD analysis and were considered for a subsequent evolution analysis. BUSTED analysis found evidence for gene-wide episodic diversifying selection at at least one site on at least one branch for both *pfhrp2* and *pfhrp3*, with a synonymous rate variation (ω) > 1. These results were confirmed by repeating the same analysis including homologous sequences from other regions of sub-Saharan Africa from GenBank.

**Fig. 5:**
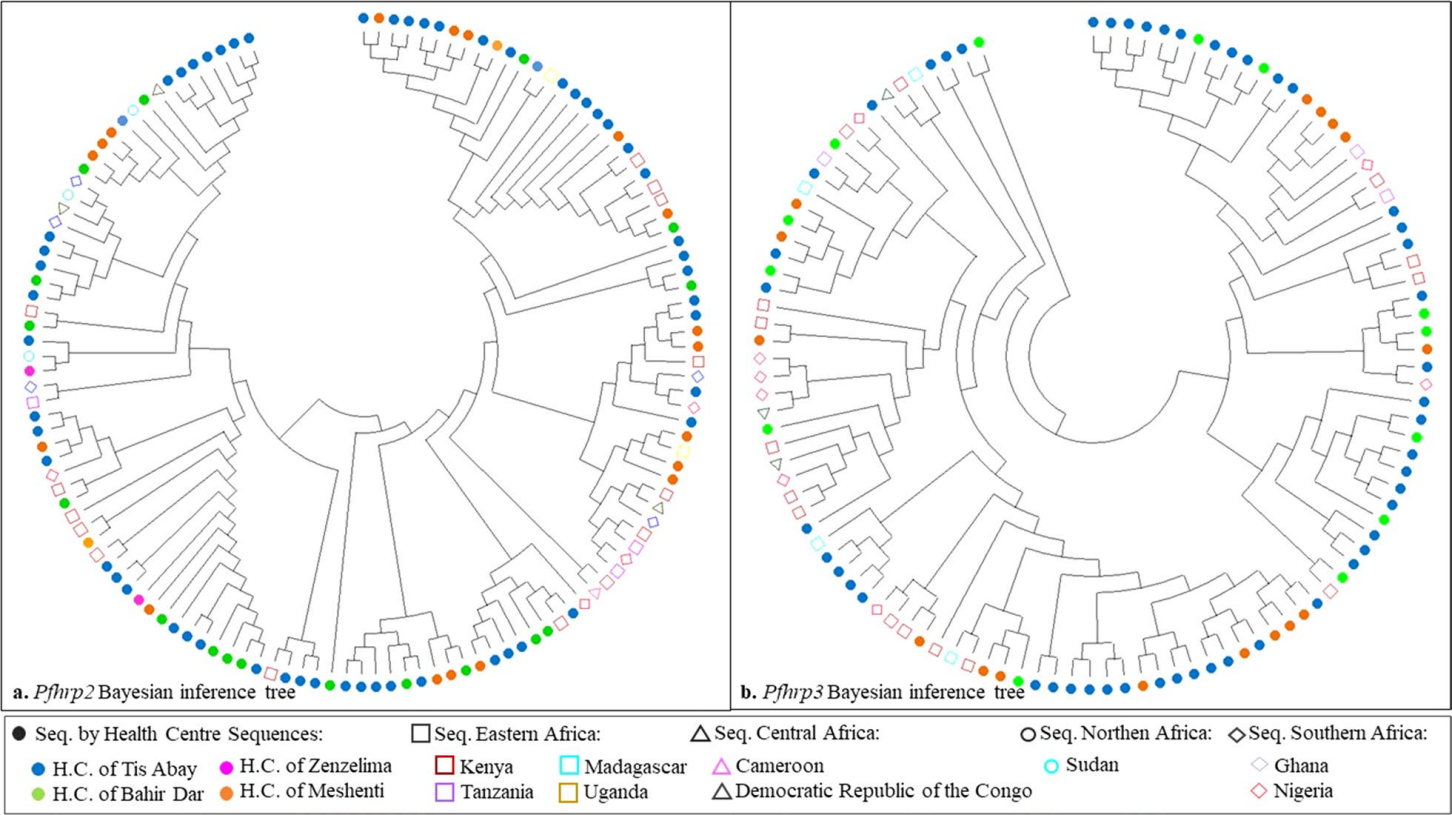
Bayesian inference phylogenetic tree for *pfhrp2* (**a**) and *pfhrp3* (**b**) sequences. The nucleotide sequences of exon 2 for both genes, with fragments ranging from 300 to 700 base pairs, were analysed. This analysis combined nucleotide sequences from this article, represented with a dot (n = 95 for *pfhrp2* and n = 79 for *pfhrp3*) and sequences from bordering countries published in previous studies, represented with dots with different shapes (n = 33 for *pfhrp2* and n = 33 for *pfhrp3*). Coloured dots indicate the geographical origin of the samples. Phylogenetic trees were performed using Bayesian inference

The Bayesian inference phylogenetic tree showed an unclear clustering by health centre, country or region for both genes (Fig. 5). The Maximum Likelihood Inference tree showed similar results. Sequences from the same health centre tend to form small clusters but without being truly isolated. Similarly, sequences from countries bordering Ethiopia (Kenya and Sudan) were more closely related to sequences from the present study. However, sequences from other African regions are also relatively closely related to some sequences, thus indicating an unclear geo-clustering.

### Variation in amino-acid repeat patterns

Differences in the frequency and organization of amino-acid repeats were detected, with the most frequent amino acids in *pfhrp2* and *pfhrp3* being alanine (37.32%), histidine (36.16%) and aspartate (9.59%), and histidine (31.18%), alanine (30.19%), asparagine (9.55%), and aspartate (9.11%), respectively.

### Variation in amino-acid repeats in *pfhrp2*

A total of 20 previously reported amino-acid repeat types were found, with repeat types 2 and 7 being observed in all isolates and with the highest average repetition frequency (type 2: 8.8-times and type 7: 4.3-times) (Table 4). The next most frequent repeats were type 3 (83%) and type 8 (90%), which appeared in the range 0–2-times per sequence, and type 4 (84%), for which the repetition times per sequence ranged from 0- to 18-times. New or modified repeat amino-acid motifs were detected and classified (Additional file 5). Organization of the amino-acid repeats in the sequences followed similar patterns, although with some variations (Additional file 6).

**Table 4:** Occurrence and number of amino-acid repeat types in *pfhrp2* and *pfhrp3* sequences

Repeat types	Amino acid repeat	Pfhrp2		Pfhrp3	
		Range of no. of repeats	Occurrence (%)	Range of no. of repeats	Occurrence (%)
Type 1	AHHAHHVAD	0–5	42	0–2	63.75
Type 2	AHHAHHAAD	1–16	**100**	0	0
Type 3	AHHAHHAAY	0–2	83	0	0
Type 4	AHH	0–18	80	0–6	88.75
Type 5	AHHAHHASD	0–2	67	0	0
Type 6	AHHATD	0–7	75	0	0
Type 7	AHHAAD	1–8	**100**	0–1	95.00
Type 8	AHHAAY	0–2	90	0	0
Type 9	AAY	0–1	3	0	0
Type 10	AHHAAAHHATD	0–3	40	0	0
Type 11	AHN	0	0	0	0
Type 12	AHHAAAHHEAATH	0–1	35	0	0
Type 13	AHHASD	0–2	18	0	0
Type 14	AHHAHHATD	0–1	13	0	0
Type 15	AHHAHHAAN	0	0	0–1	68.75
Type 16	AHHAAN	0–1	1	0–17	98.75
Type 17	AHHDG	0	0	0–8	90.00
Type 18	AHHDD	0	0	0–4	88.75
Type 19	AHHAA	0–2	23	0–1	1.25
Type 20	SHHDD	0	0	0–1	87.5
Type 21	AHHAHHATY	0	0	0	0
Type 22	AHHAHHAGD	0	0	0	0
Type 23	ARHAAD	0	0	0	0
Type 24	AHHTHHAAD	0	0	0	0

### Variation in amino-acid repeats in *pfhrp3*

A total of 15 previously reported amino-acid repeat types were found in the sequences (Table 4). Repeat type 16 was included in the majority of isolates (99%) and was the most repeated type in the sequences (average time: 11.15). The following more frequent types had markedly fewer repeats per sequence. For example, types 7 and 20 were found in 95% and 88% of samples, respectively, but only one time per sequence. The organization of amino-acid repeats was similar for all samples and in agreement with previously reported results (Additional file 7).

### Structure prediction and epitope localization

#### In silico structure prediction

The secondary and tertiary protein structure was predicted for a sub-set of samples (n = 22), selected based on sequence quality, using RaptorX. For most samples, the majority of the secondary structure was a helix, with some coils placed principally in intermediate positions. Batch Phyre2 structure-based function prediction analyses were carried out for 96 samples, with a significant result being found for only 10 samples: significant results are needed for structure prediction and homology. Among these, the main structure matches were with proteins with a hydrolase function (Additional file 8).

#### Epitopes of HRP2 and HRP3 targeted by monoclonal antibodies

Seven of the 11 major antibody epitopes were identified in more than 84% of *pfhrp2* sequences (n = 81) (Table 5). Two epitopes, namely 3A4 and C1-13, were present in all sequences with the highest median frequencies: 14- and 13-times per sequence, respectively. Epitopes 2G12-1C12, N7 and PTL-3 were present in 99, 99 and 95% of sequences, respectively, but at lower frequencies (2–4-times per sequence).

**Table 5:** Frequency of major epitopes in *pfhrp2* and *pfhrp3* exon-2 targeted by MAbs in HRP2-based RDT

MAb	Major epitope	*Pfhrp2*		*Pfhrp3*	
		Frequency (%)	Median frequency (n)	Frequency (%)	Median frequency (n)
3A4	AHHAHHA	100	14	70	1
2G12-1C12	DAHHAADAHH	99.06	4	0	0
1E1-A9	AHHAHHV	42.06	0	65	1
A6-4	HATDAHH	84.11	3	0	0
C1-13	AHHAADAHH	100	13	4	0
N7	DAHHAADAHHA	99.06	4	0	0
PTL-3	YAHHAHHA	95.33	2	0	0
S2-5	AHHASDAHHA	84.11	1	0	0
TC-10	TDAHHAADAHHAADA	54.21	1	0	0
C2-3	HAHHAHHAADAHH	43.93	0	0	0
Genway	AYAHHAHHAAY	0	0	0	0

Among *pfhrp3* sequences, epitope 3A4 was the most common (70%), but with only a low frequency (median = 1), followed by 1E1-A9, which was found in 65% of sequences (median = 1).

**Fig. 6:**
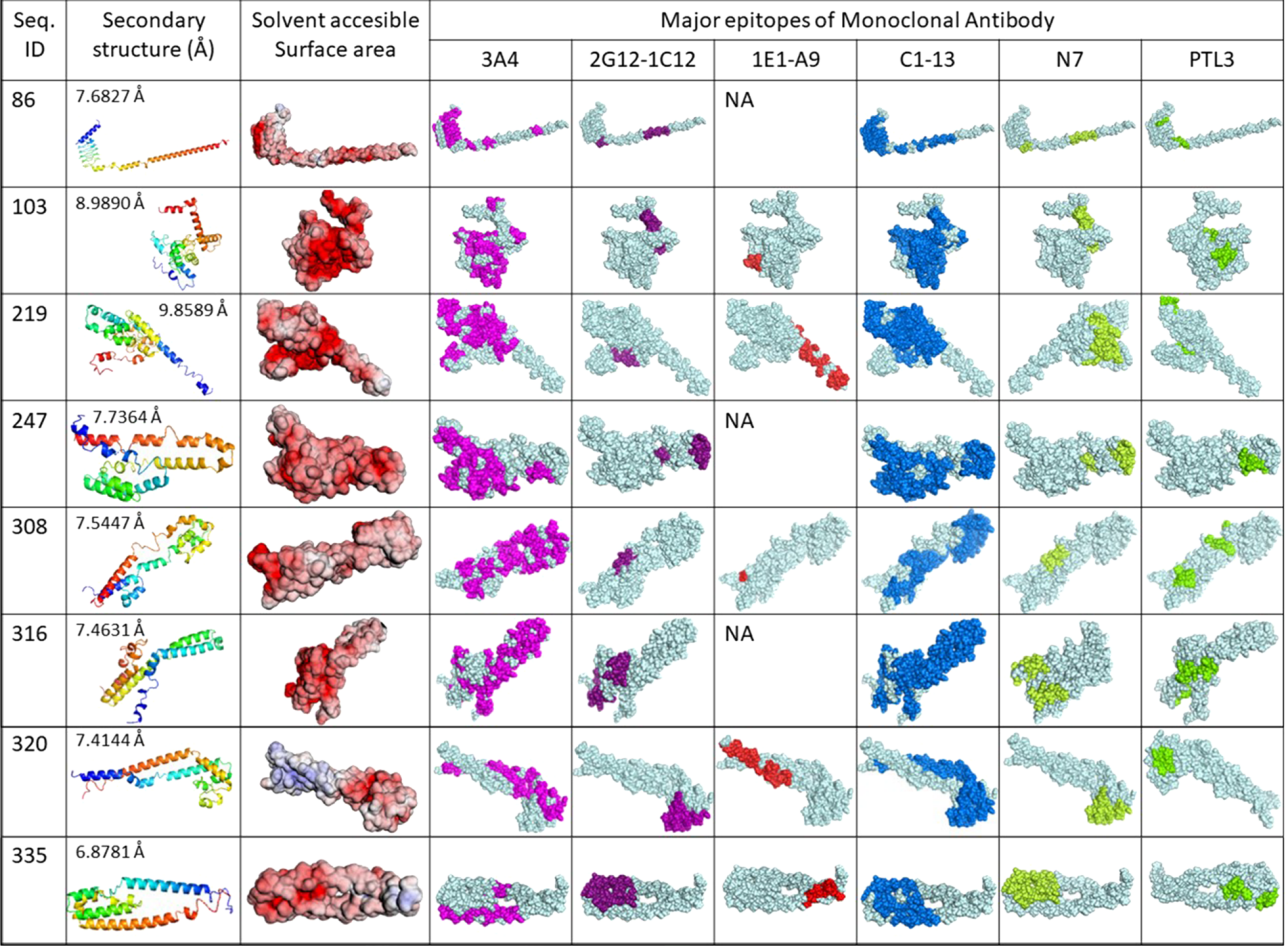
Secondary structure, solvent surface area and mapping of epitopes in the PfHRP2 protein structure models. Model of structure was assessed by RSMD measured with Å. Solvent surface area is coloured gradually according to electrostatic potential from − 5 kT/e (red) to + 5 kT/e (blue). NA: Not applicable due to absence of epitope in the sequence

**Fig. 7:**
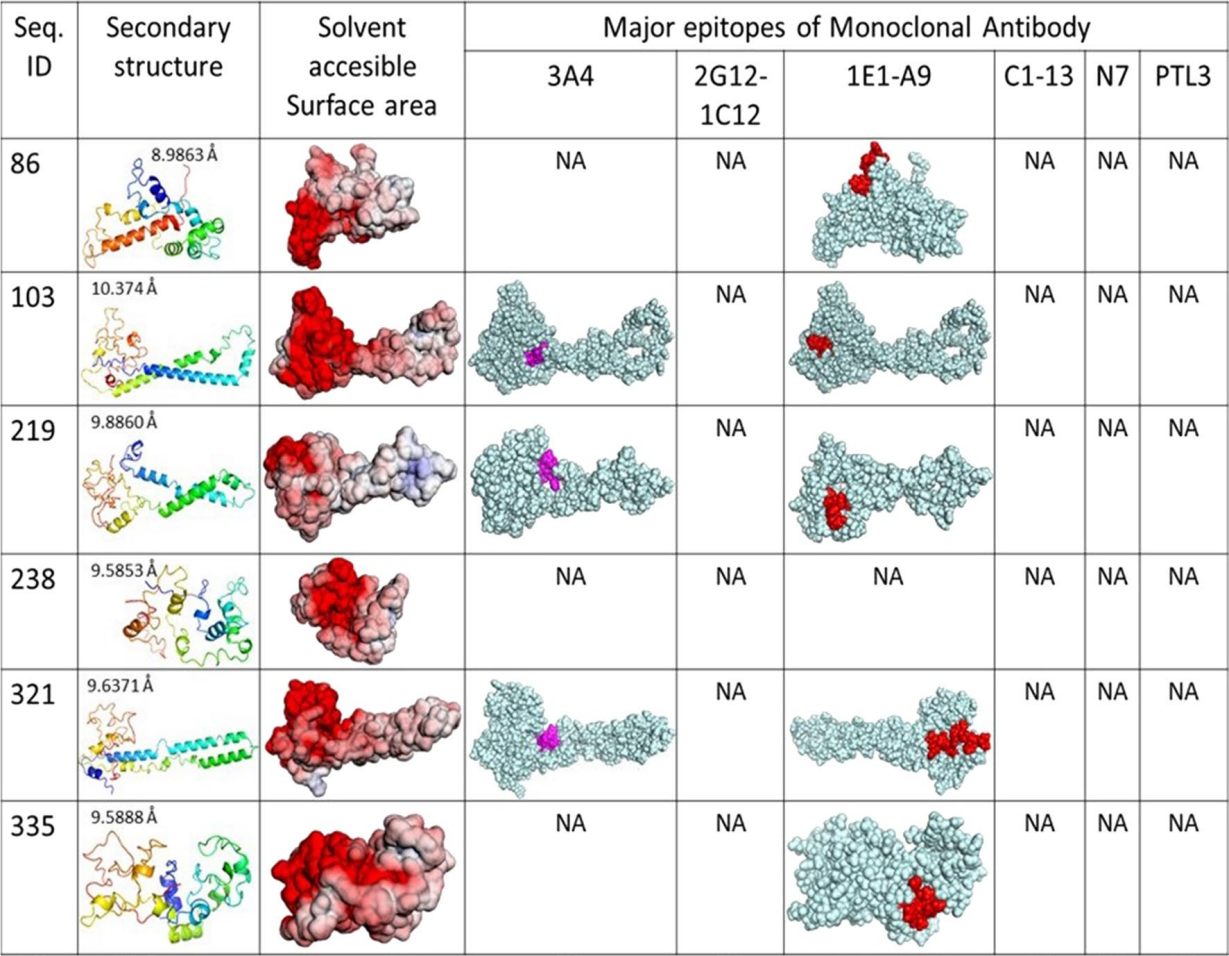
Secondary structure, solvent surface area and mapping of epitopes in the PfHRP3 protein structure models. Model of structure was assessed RSMD measured with Å. Solvent surface area is coloured gradually according to electrostatic potential from − 5 kT/e (red) to + 5 kT/e (blue). NA: Not applicable due to absence of epitope in the sequence

The eight tertiary structure models were used to locate the main epitopes in the structure. These were then compared with the solvent-accessible surface area, which has a mainly negative electrostatic potential. Epitopes 3A4 and C1-13 were found to be the most accessible as they were widely distributed over the protein surface, whereas other epitopes were located in only a specific region of the surface (Fig. 6). Additionally, epitopes recognized by 3A4 were also found in the PfHRP3 models studied, but with less accessibility (Fig. 7).

## Discussion

An understanding of *pfhrp2* and *pfhrp3* gene deletions and genetic diversity is key to the design of future interventions in RDT-based malaria test-and-treat strategies. This study describes the frequency of deletions and unique data regarding the sequence variation of *pfhrp2* and *pfhrp3* from the Amhara region in Ethiopia.

### *pfhrp2 *and *pfhrp3* deletions

This study showed that the detected frequency of the exon-2 deletion for *pfhrp2* in the Amhara region of Ethiopia (4.64%) is worryingly close to the threshold (5% at the lower end of the 90% CI for false negatives by HRP2-based RDT) established by the WHO to change the use of HRP2-based RDTs [46]. Although this study did not use results from RDT, a higher prevalence of deletion among HRP2-based RDT false-negatives than for samples diagnosed by microscopy, where false negatives and true positives by HRP2-based RDT could be mixed, is to be expected. Taking this into account, the higher frequency of deletion (11.5%) reported for RDT false-negatives in 2018 in Amhara suggests a gradual increase [22]. In contrast, a higher frequency of deletion has been reported in other regions of Ethiopia, for example 18% among samples including true positives and false negatives and 42% among HRP2-based RDT false-negatives in the Assosa region, with high/moderate transmission [29], and 100% among general samples in Oromia, with low transmission [30]. The differences between reported prevalence could be related to the association between deletion frequency and low malaria transmission, where it is less probable to find a mixed infection of parasites with and without *pfhrp2* that masks the deletion [20]. Another explanation for the wide range of deletion prevalence reported could be the differences in genotyping approach, for example, a recent study in Ethiopia only analysed samples with low or without HRP2 signal by multiplex bead assay [47]. That makes it essential to generalized technical strategies across studies [48]. Potential false negatives caused by *pfhrp2* deletions could be avoided by cross-reaction with pfHRP3 as this can be detected by HRP2-based RDT in the absence of pfHRP2 [4]. However, in the light of this study’s results, in Ethiopia this is unlikely to be possible due to the high frequency of *pfhrp3* deletions (18.5%) and isolates with deletions in both genes.

With regard to patterns of deletions, the findings are in agreement with the literature, which shows that deletions in exon 1–2 and in *pfhrp3* are more common than in exon 2 and in *pfhrp2*, respectively [49, 50]. This may suggest the possibility of sequential deletion, starting with deletion in exon 1–2 but ending with complete deletion [51]. But, deletions in only one exon, called in this manuscript ‘candidate partial deletion’, could not be confirmed and could be simply due to worse amplification in exon 1–2. However, deletions in different fragments of these genes have also been reported, suggesting that the full gene is not always deleted [22]. Evolution and selection of *pfhrp* gene deletions will be closely associated with their fitness cost [52]. Although this remains to be clarified, previous studies in vitro have suggested the selection of strains without deletions and slower growth in strains with deletions [21, 53]. Moreover, in Ethiopia the extended used of combination HRP2/Pv or Pan—LDH-based RDT [54], due to high prevalence of *P. vivax*, could weaken the selection pressure of *pfhrp*-deleted parasites, unlike in other sub-Saharan countries.

### Genetic diversity and evolution analysis of exon 2 from *pfhrp2* and *pfhrp3* genes

The dynamics of the spread or selection of *pfhpr2*-deleted populations remains unclear. However, as HRP2-based RDT use has increased exponentially in recent years, a selective pressure for parasites with *pfhrp2* deletions could be expected [31]. Indeed, linkage-specific clonal expansion of populations with *pfhrp2* deletions has been reported [55]. In contrast, the results of the BUSTED test showed diversifying expansion in the present study. This diversity, combined with a lack of clear clustering, could be related to an increased diversity step, when gene deletions occur, followed by clonal expansion promoted by selective pressure. In fact, recent strong selection of *pfhrp2*-deleted parasites have been reported from Ethiopia in a study carried out some years later, when this clonal expansion, boosted by test-track-treat policies, could be expected to happen [22]. Multiple spontaneous origins of deletions in these genes, which could be part of the diversifying expansion, have been reported previously [22, 56].

### Variation in amino-acid repeat patterns and its implication for the effectiveness of HRP2-based RDT

The role of a variation in amino-acid repeats in RDT is unclear. Previous studies have reported both an influence on RDT results, increasing its sensitivity or causing misdiagnosis [57, 58], or a lack of association [59]. Parasites could maintain the gene but undergo changes in some specific amino-acid repeats, thereby becoming undetectable [60, 61]. Some repeat types could have more influence in HRP2-based RDT results, but there is not found a clear association [13]. This suggests the need to monitor the effectiveness of RDT.

The *pfhrp2* sequences showed 16 previously described repeat types, including some variations. The recent increase in reported variations and new domains is consistent with a diversifying expansion [40, 62]. In line with other studies, the most common repeats were types 2 and 7, the frequency of which markedly influences RDT detection [15, 63].

*Pfhrp3* sequences showed less diversity in terms of amino-acid sequences than *pfhrp2*, as reported previously [60]. Similar to previous studies, the most frequent and repeated type was type 16 [64, 65], and type 17 showed a marked variation, as in Kenya [61], thus suggesting a high antigenicity and that it may be useful for RDT detection.

### Protein structure prediction and epitope localization

The relative similarity between the occurrence and organization of amino-acid repeats, either with geographically close parasites or those from other continents, contrasts with the high diversity detected at a nucleotide level. However, this could imply the temporal persistence of PfHRP2 epitopes despite the genetic diversity [61]. For instance, the results from the Amhara region are similar to those reported previously in other countries for the 12 available PfHRP2-specific MAbs. Although information about the antibodies used in commercial RDTs is unavailable, it is reasonable to assume that many of these epitopes are targeted [17]. The epitopes recognized by MAbs 3A4 and C1-13 were the most frequent [62, 66], and the epitope for 3A4 was also the most common in *pfhpr3* sequences [24]. Additionally, the epitope recognized by C1-13 was identified for the first time in the *pfhrp3* sequence in this paper. This detection of the usual epitopes increases the probability of parasite detection, even though they were present at only low frequencies.

Protein-structure models showed broad surfaces with solvent accessible surface areas on which the majority of linear epitopes were found and shown to be topographically available. In contrast, few of the linear epitopes were detected among HRP3 protein sequences, thus suggesting that, in this protein, the epitopes recognized by HRP2-RDT could be conformational rather than linear.

*Pfhrp2* and *pfhrp3* deletions might have a different impact depending on malaria endemicity. Low malaria diversity settings, which are related to a low prevalence of malaria, could lead to an easier selection of deleted parasites [67]. As both the competition and recombination between different strains of parasites are lower, deletion will be easier to maintain in following generations. The exception to this is parasites with both anti-malarial resistance and deletions, as they could be more easily selected irrespective of the conditions [20]. As such, surveillance of *pfhrp2* and *pfhrp3* is particularly important in settings in which malaria is decreasing as a result of prevention and control efforts, such as Ethiopia [68, 69]. Although HRP2-based RDTs are still considered useful for malaria diagnosis in endemic regions [70], WHO guidance recommends countrywide change when the threshold was exceeded in any region. Meanwhile, combination of pLDH-RDTs with targets other than HRP2 are preferable [9, 71].

There are some limitations to this study. For example, samples were collected based on microscopy smears positive for *P. falciparum* but RDT results or parasite densities were not available, thus limiting the power to detect the effect of deletions in malaria diagnosis. Furthermore, representativeness and comparisons between the different health centres was not possible epidemiologically due to the low number of samples. Also, candidate partial deletions could not be confirmed by genetic analysis. Moreover, genetic analysis of repetitive genes required the exclusion of large number of sites affecting genetic diversity results. However, the study still provides a more in-depth genetic characterization of *pfhrp2* and *pfhrp3* genes and their amino-acid composition. Subsequent studies should assess the implications of variations in sequences or epitopes and their effect on RDT results. Also, full-length of ‘partially deleted’ samples will provide insightful information about this partial deletions.

## Conclusion

*Pfhrp2* and *pfhrp3* deletions have been identified in *P. falciparum* isolates from Amhara region in Ethiopia. The high frequency of exon 2 *pfhrp2* deletion and the detection of isolates with double deletions in both exon 2 of *pfhrp2* and *pfhrp3* could jeopardize malaria-control strategies based on diagnosis with HRP2-based RDTs.

The high genetic diversity and high polymorphism of the amino-acid sequences found in *pfhrp2* and *pfhrp3* may have an impact on RDT results and malaria diagnosis, especially given the relatively low frequency of repetition types 2 and 7 detected.

This study also provides insights into the localization of epitopes in the protein structure of *pfhrp2* and *pfhrp3*, with the use of combinations of MAbs 3A4 and C1-13 being recommended to increase the reactivity of HRP2-based RDTs. However, a better understanding of protein structure could be useful for the design and evaluation of monoclonal antibodies for RDT.

In light of all these results and the malaria situation in Ethiopia, this study highlights the importance of more surveillance studies to increase our understanding of the dynamics of these genes.

## Supplementary Information

**Additional file 1: Table S1.** Origin of homologous sequences included for phylogenetic analysis.

**Additional file 2.** Custom code used in Python for analysis of aminoacidic repetition types.

**Additional file 3: Table S2.** Frequencies of deletion patterns of exon 2 of pfhrp2 and pfhrp3 genes by location.

**Additional file 4: Table S3.** Haplotypes frequency of not—unique samples in pfhrp2 and pfhrp3 by sampling located in Ahmara region. Figure S1. Haplotype networks of pfhpr2 (a) and pfhrp3 (b). Colour represents different sample The size of the circles are proportional to the number of sequences include in each haplotype.

**Additional file 5: Table S4.** Occurrence and number of variations of Baker’s amino acid repeats types in pfhrp2 and *pfhrp3 *sequences. *Amino acid repeats types described by Nderu et al. [61]. ^New amino acid repeats types

**Additional file 6: Figure S2.** Structural organisation of pfhrp2 amino acid repeats types. Type 1, when is present, appeared always at the beginning of the sequence with 1 to 5 consecutives repeats following by various consecutive type 2 repeats. In the middle of the sequences, there were different numbers of consecutive type 2 repeats usually mixed with type 4, type 5, type 6, type 7 and type 8, but there was not any clear pattern. At the end of the sequence, usually appeared a unique type 12 repeat, preceded by type 10 repeat.

**Additional file 7: Figure S3.** Structural organisation of pfhrp3 amino acid repeats types. The most common organisation of repeats started with one repeat type 1 followed by one repeat type 15 and around 10 consecutive repeats of type 16 ending with one repeat of type 7. Then, after a space of 33 aa without classified repeats, there were interspersed repeats of type 17, more abundant, and type 18. It ends with two repeats of type 4.

**Additional file 8: Figure S4.** Tertiary protein structure models according to template-based prediction.

## Data Availability

The datasets supporting the conclusions of this article are included within the article and its additional files, and the databases can be made available upon request to qualified researchers.
