# Supplementary material for: Deletion patterns, genetic variability and protein structure of pfhrp2 and pfhrp3: implications for malaria rapid diagnostic test in Amhara region, Ethiopia
Source: Malar J. 2022 Oct 8;21:287. doi: 10.1186/s12936-022-04306-3 (PMC9548178; doi:10.1186/s12936-022-04306-3)
Supplement: Supplementary file 1 — Additional file 1: Table S1. Origin of homologous sequences included for phylogenetic analysis. [file 12936_2022_4306_MOESM1_ESM.pdf]

Additional file 1.

Table S1. Origin of homologous sequences included for phylogenetic analysis.

**Country**	**Nº of sequences included**	**GenBank Accession number**	**Reference**
Pfhrp2 sequences			
Kenya	9	MH230284, MH230285, MH230385, MH230355, MH230395, MH230455, MH230468, MH230510, MH230520	(Nderu, Kimani, Thiong'o, et al., 2019)
Sudan	3	AY816237, AY816238, AY816239	(J. Baker et al., 2005)
Cameroon	2	AY816249, AY816250	
Ghana	2	AY816251, AY816262	
Uganda	1	AY816271	
Kenya	4	FJ871188, FJ871189, FJ871203, FJ871216	(J. Baker et al., 2010)
Nigeria	4	FJ871260, FJ871261, FJ871263, FJ871270	
Democratic Republic of Congo	3	FJ871180, FJ871181, FJ871179	
Ghana	1	FJ871217	
Tanzania	3	FJ871373, FJ871374, FJ871375	
Uganda	1	FJ871400	
Pfhrp3 sequences			
Kenya	11	MH230527, MH230540, MH230543, MH230575, MH230580, MH230584, MH230588, MH230592, MH230596, MH2305600, MH230601	(Nderu, Kimani, Thiong'o, et al., 2019)
Democratic Republic of Congo	3	GU194978, GU194980, GU194981	(J. Baker et al., 2010)
Kenya	4	GU194977, GU194982, GU194983, GU194984	
Nigeria	8	GU194993, GU194994, GU194995, GU194996, GU194997, GU194998, GU195016, GU195036	
Madagascar	4	GU194999, GU195003, GU195004, GU195005	
Tanzania	3	GU195039, GU195040, GU195041	
