# Supplementary material for: Deletion patterns, genetic variability and protein structure of pfhrp2 and pfhrp3: implications for malaria rapid diagnostic test in Amhara region, Ethiopia
Source: Malar J. 2022 Oct 8;21:287. doi: 10.1186/s12936-022-04306-3 (PMC9548178; doi:10.1186/s12936-022-04306-3)
Supplement: Supplementary file 2 — Additional file 2. Custom code used in Python for analysis of aminoacidic repetition types. [file 12936_2022_4306_MOESM2_ESM.pdf]

Additional file 2.

Custom code used in Python for analysis of aminoacidic repetition types

```
#Aminoacidic repetition type
string = ""
pattern = ""
def PatternCount(string, pattern):
    count = 0
    for i in range(len(string)-len(pattern)+1):
        if string[i:i+len(pattern)] == pattern:
            count=count+1
    return count
print (PatternCount(string, pattern))
def PatternMatch(pattern, string):

    record= []
    for i in range(len(string)-len(pattern)):
        if pattern == string[i:i+len(pattern)]:
            record.append(i)
    return record
print (PatternMatch(pattern, string))
```
