# Supplementary material for: Deletion patterns, genetic variability and protein structure of pfhrp2 and pfhrp3: implications for malaria rapid diagnostic test in Amhara region, Ethiopia
Source: Malar J. 2022 Oct 8;21:287. doi: 10.1186/s12936-022-04306-3 (PMC9548178; doi:10.1186/s12936-022-04306-3)
Supplement: Supplementary file 3 — Additional file 3: Table S2. Frequencies of deletion patterns of exon 2 of pfhrp2 and pfhrp3 genes by location. [file 12936_2022_4306_MOESM3_ESM.pdf]

Additional file 3

Table S2. Frequencies of deletion patterns of exon 2 of pfhrp2 and pfhrp3 genes by location.

Location	*pfhrp2* deletion			*pfhrp3* deletion		
	N total	P (90% IC)	n. of isolates	N total	P (90% IC)	n. of isolates
Tis Abay	183	7.10 (4.25 – 11.06)	13	173	17.92 (13.28 – 23.41)	31
Bahir Dar	54	7.41 (2.57 – 16.15)	4	43	16.28 (7.90 – 28.41)	7
Zenzelima	11	18.18 (3.33 – 47.00)	2	8	75.00 (40.03 – 95.36)	6
Meshenti	32	3.13 (16.02 – 13.98)	1	26	7.69 (1.38 – 22.29)	2
p-value	0.529			< 0.001		
