# Supplementary material for: Deletion patterns, genetic variability and protein structure of pfhrp2 and pfhrp3: implications for malaria rapid diagnostic test in Amhara region, Ethiopia
Source: Malar J. 2022 Oct 8;21:287. doi: 10.1186/s12936-022-04306-3 (PMC9548178; doi:10.1186/s12936-022-04306-3)
Supplement: Supplementary file 4 — Additional file 4: Table S3. Haplotypes frequency of not—unique samples in pfhrp2 and pfhrp3 by sampling located in Ahmara region. Figure S1. Haplotype networks of pfhpr2 (a) and pfhrp3 (b). Colour represents different sample The size of the circles are proportional to the number of sequences include in each haplotype. [file 12936_2022_4306_MOESM4_ESM.pdf]

Additional file 4

Table S3. Haplotypes frequency of not – unique samples in pfhrp2 and pfhrp3 by sampling location in Ahmara region.

Pfhrp2						***Pfhrp3***				
**Hap.**	**Frequency (%)**					**Hap.**	**Frequency (%)**			
	Overall N = 96	Tis Abay N= 55	Bahir Dar N = 13	Zenzelima N = 3	Meshenti N = 25		Overall N= 78	Tis Abay N= 52	Bahir Dar N = 10	Meshenti N = 16
**8**	2 (2.08%)	2 (3.64%)	0	0	0	**16**	2 (2.56%)	1 (1.92%)	1 (10%)	0
**14**	2 (2.08%)	1 (1.82%)	1 (7.69%)	0	0	**32**	2 (2.56%)	1 (1.92%)	1(10%)	0
**15**	2 (2.08%)	2 (3.64%)	0	0	0					

Figure S1. Haplotype networks of pfhpr2 (a) and pfhrp3 (b). Colour represents different sample origin. The size of the circles are proportional to the number of sequences include in each haplotype.

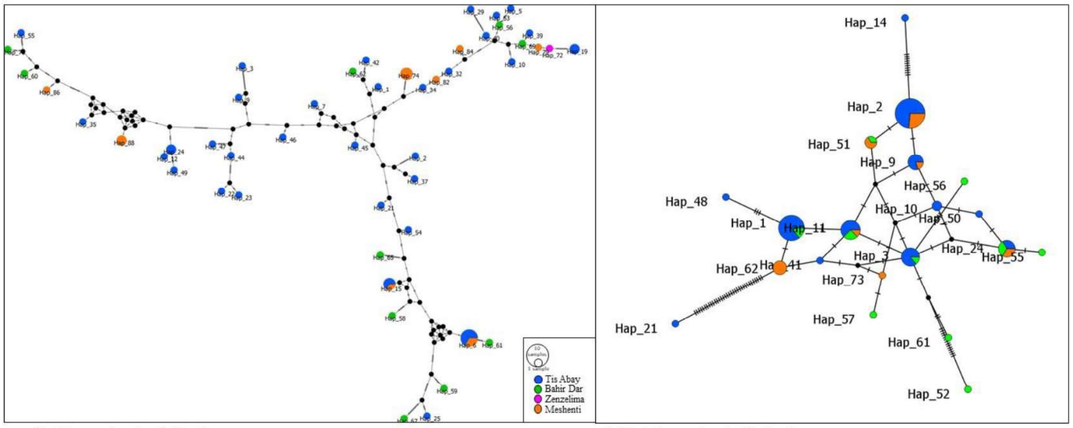

a. Haplotype network of *pfhrp2* **b.** Haplotype network of *pfhrp3*
