# Supplementary material for: Deletion patterns, genetic variability and protein structure of pfhrp2 and pfhrp3: implications for malaria rapid diagnostic test in Amhara region, Ethiopia
Source: Malar J. 2022 Oct 8;21:287. doi: 10.1186/s12936-022-04306-3 (PMC9548178; doi:10.1186/s12936-022-04306-3)
Supplement: Supplementary file 5 — Additional file 5: Table S4. Occurrence and number of variations of Baker’s amino acid repeats types in pfhrp2 and pfhrp3 sequences. *Amino acid repeats types described by Nderu et al. [61]. ^New amino acid repeats types [file 12936_2022_4306_MOESM5_ESM.pdf]

Additional file 5

Table S4. Occurrence and number of variations of Baker's amino acid repeats types in pfhrp2 and *pfhrp3* sequences. *amino acid repeats types described by Nderu et al. (2018). ^New amino acid repeats types.

About *pfhrp2* it is highlighted the appearance of the motif "AHHAADD" in 4 sequences, however it was not classified as new domain as it was count as type 7 repeat variation. Also, type 11 was only found with the last amino acid modified (AHQ). AHVDD and AHHVAD, considered new repeats, and AHHAHHAVD, considered modification of type 1 repeat, were placed at the beginning of sequences. The most highlighted modification found in *pfhrp3* was Type 11, that was only present modified (AHQ) and in two parasites.

		Pfhrp2		***Pfhrp3***	
Repeat types	Amino acid repeat	Range of nº of repeats	Occurrence (%)	Range of nº of repeats	Occurrence (%)
Type 1	AHHAHYVAD*	0	0	0	0
	AHHAHHAVD^	0 - 1	2	0	0
Type 2	AHHAHHADD^	0-2	3	0	0
Type 4	ASH*	0 – 2	3	0 - 2	3.75
	THH*	0 – 1	4	0 – 3	51.25
	AAH*	0	0	0 - 1	1.25
	APH^	0 – 4	8	0 - 2	2.5
	ADH^	0 – 1	4	0 - 1	1.25
	VHH	0	0	0 – 1	3.75
Type 5	AHHAHHAPD*	0 - 2	7	0	0
	AHHAHHAYD^	0 - 2	2	0	0
Type 6	APHATD*	0	0	0	0
Type 7	AHHAPD*	0 - 1	6	0	0
	ALHAAD^	0 - 1	1	0	0
	AHHAVD^	0 - 1	3	0	0
Type 8	AHHASY*	0 - 1	1	0	0
Type 10	AHHAATHHATD*	0	0	0	0
Type 11	AHQ^	0 - 1	8	0 – 1	2.5
Type 12	AHHAAAHHEAATP*	0	0	0	0
	AHHASAHHEAATH^	0 - 1	1	0	0
	AHHAAAHHEDATH^	0 – 1	1	0	0
	AHHAPAHHEAATH^	0 - 1	1	0	0

Type 16	AHHAAK*	0	0	0	0
	SHHAAN*	0	0	0	0
	DHHAAN^	0	0	0 – 4	1.25
	APHAAN^	0	0	0 – 1	1.25
	APNAAN^	0	0	0 – 1	1.25
	AHHASN^	0	0	0 – 1	1.25
Type 17	QHHDG*	0	0	0	0
	SHHDG*	0	0	0 - 1	7.5
	THHDG*	0	0	0 - 1	1.25
	AHHDE*	0	0	0 - 1	5
Type 18	EHHDD*	0	0	0	0
New repeats	AHVDD^	1	22	0	0
	AHHAPH^	1 - 4	7	0	0
	AHHVAD^	0 -1	12	0 - 1	10
