# Supplementary material for: Deletion patterns, genetic variability and protein structure of pfhrp2 and pfhrp3: implications for malaria rapid diagnostic test in Amhara region, Ethiopia
Source: Malar J. 2022 Oct 8;21:287. doi: 10.1186/s12936-022-04306-3 (PMC9548178; doi:10.1186/s12936-022-04306-3)
Supplement: Supplementary file 6 — Additional file 6: Figure S2. Structural organisation of pfhrp2 amino acid repeats types. Type 1, when is present, appeared always at the beginning of the sequence with 1 to 5 consecutives repeats following by various consecutive type 2 repeats. In the middle of the sequences, there were different numbers of consecutive type 2 repeats usually mixed with type 4, type 5, type 6, type 7 and type 8, but there was not any clear pattern. At the end of the sequence, usually appeared a unique type 12 repeat, preceded by type 10 repeat. [file 12936_2022_4306_MOESM6_ESM.pdf]

Additional file 6.

Figure S2. Structural organisation of pfhrp2 amino acid repeats types. Type 1, when is present, appeared always at the beginning of the sequence with 1 to 5 consecutives repeats following by various consecutive type 2 repeats. In the middle of the sequences, there were different numbers of consecutive type 2 repeats usually mixed with type 4, type 5, type 6, type 7 and type 8, but there was not any clear pattern. At the end of the sequence, usually appeared a unique type 12 repeat, preceded by type 10 repeat.

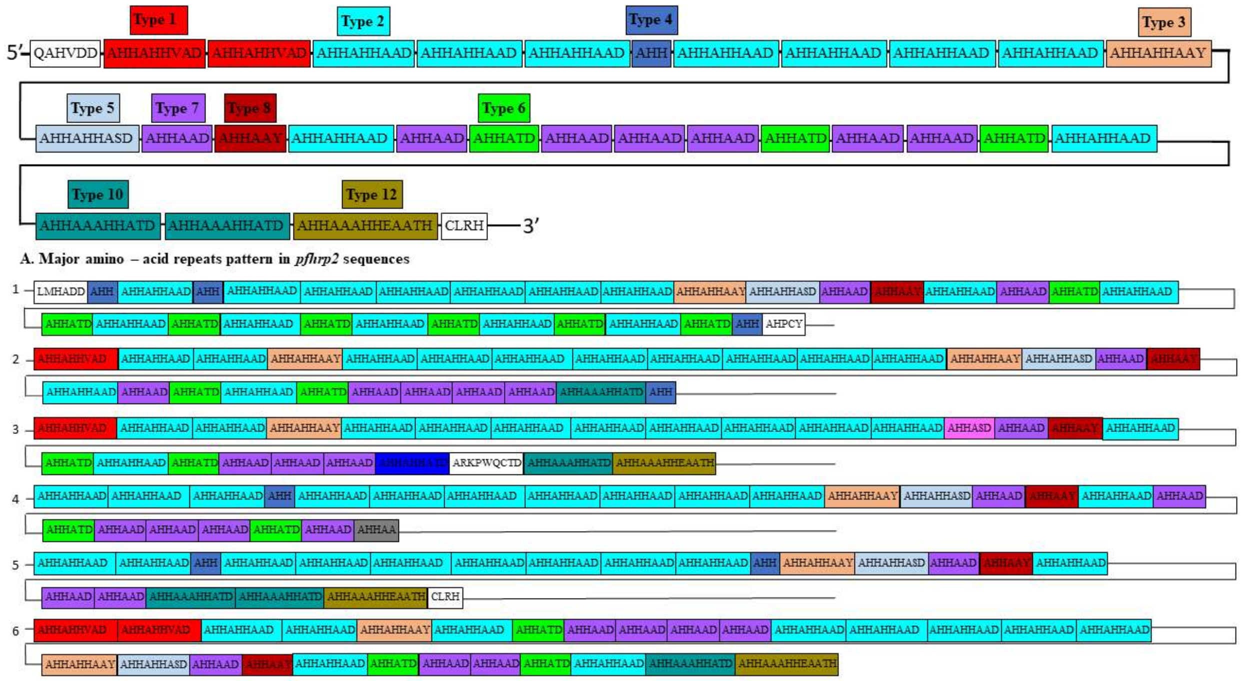

B. Other amino – acid repeats pattern in *pfhrp2* sequences
