# Supplementary material for: Deletion patterns, genetic variability and protein structure of pfhrp2 and pfhrp3: implications for malaria rapid diagnostic test in Amhara region, Ethiopia
Source: Malar J. 2022 Oct 8;21:287. doi: 10.1186/s12936-022-04306-3 (PMC9548178; doi:10.1186/s12936-022-04306-3)
Supplement: Supplementary file 7 — Additional file 7: Figure S3. Structural organisation of pfhrp3 amino acid repeats types. The most common organisation of repeats started with one repeat type 1 followed by one repeat type 15 and around 10 consecutive repeats of type 16 ending with one repeat of type 7. Then, after a space of 33 aa without classified repeats, there were interspersed repeats of type 17, more abundant, and type 18. It ends with two repeats of type 4. [file 12936_2022_4306_MOESM7_ESM.pdf]

**Additional file 7.**

Figure S3. Structural organisation of pfhrp3 amino acid repeats types. The most common organization of repeats started with one repeat type 1 followed by one repeat type 15 and around 10 consecutive repeats of type 16 ending with one repeat of type 7. Then, after a space of 33 aa without classified repeats, there were interspersed repeats of type 17, more abundant, and type 18. It ends with two repeats of type 4.

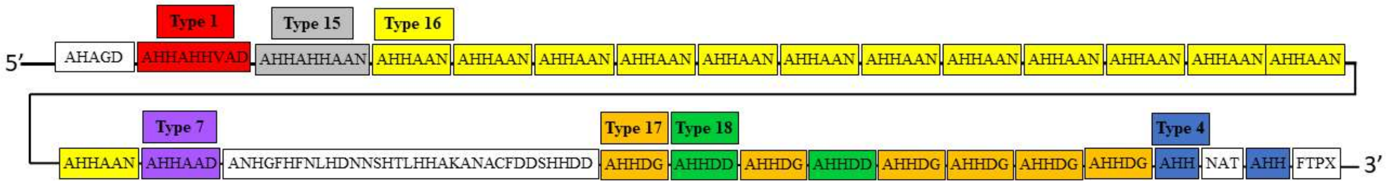


**A. Major amino – acid repeats pattern in *pfhrp3* sequences**

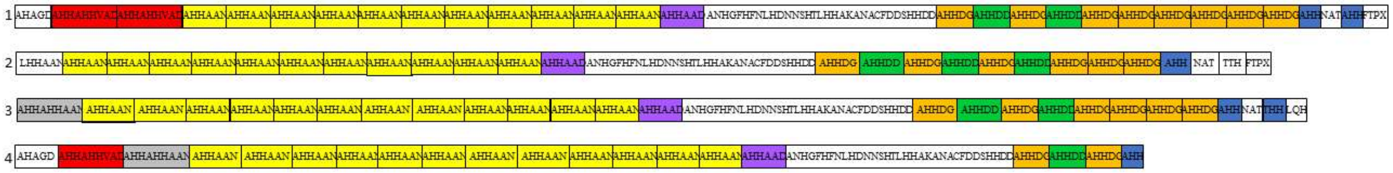

**B. Other amino – acid repeats pattern in *pfhrp3* sequences**
