# Supplementary material for: Deletion patterns, genetic variability and protein structure of pfhrp2 and pfhrp3: implications for malaria rapid diagnostic test in Amhara region, Ethiopia
Source: Malar J. 2022 Oct 8;21:287. doi: 10.1186/s12936-022-04306-3 (PMC9548178; doi:10.1186/s12936-022-04306-3)

Additional file 8.

Figure S4. Tertiary protein structure models according to template – based prediction.

Confidence: Probability of a true homology in the match between the sequence and the template
%id: Percentage of identity between the sequence and the template

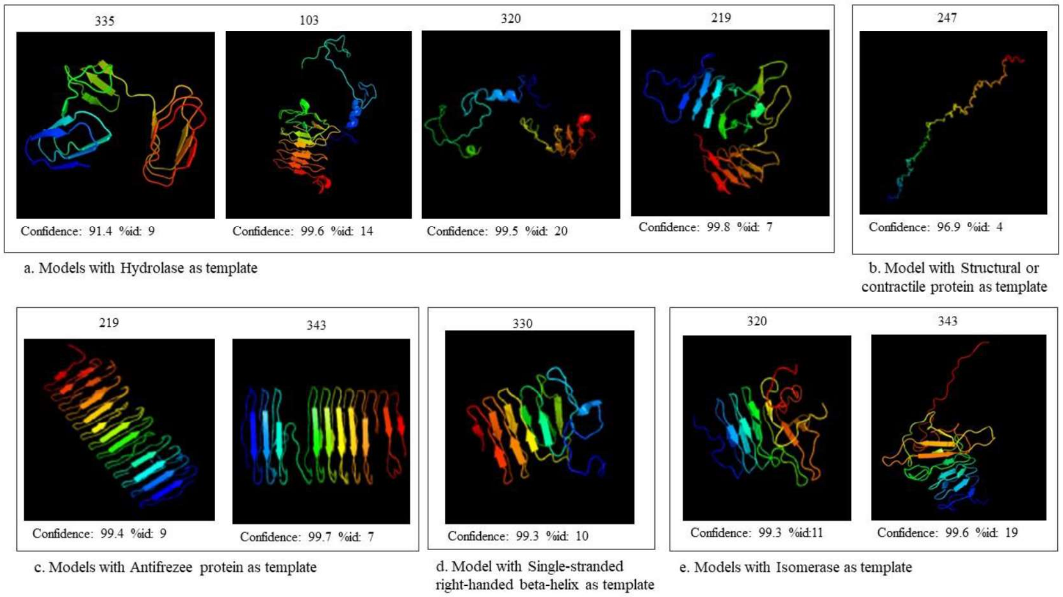

Supplement: Supplementary file 8 — Additional file 8: Figure S4. Tertiary protein structure models according to template-based prediction. [file 12936_2022_4306_MOESM8_ESM.pdf]
